# High Plains wheat mosaic virus: An enigmatic disease of wheat and corn causing the High Plains disease

**DOI:** 10.1111/mpp.13113

**Published:** 2021-08-10

**Authors:** Satyanarayana Tatineni, Gary L. Hein

**Affiliations:** ^1^ USDA‐ARS and Department of Plant Pathology University of Nebraska‐Lincoln Lincoln Nebraska USA; ^2^ Department of Entomology University of Nebraska‐Lincoln Lincoln Nebraska USA

**Keywords:** barley, cultural practice, *Emaravirus*, High Plains virus, High Plains wheat mosaic virus, maize, negative‐sense RNA virus, wheat, wheat curl mite, wheat mosaic virus

## Abstract

**Brief history:**

In 1993, severe mosaic and necrosis symptoms were observed on corn (maize) and wheat from several Great Plains states of the USA. Based on the geographical location of infections, the disease was named High Plains disease and the causal agent was tentatively named High Plains virus. Subsequently, researchers renamed this virus as maize red stripe virus and wheat mosaic virus to represent the host and symptom phenotype of the virus. After sequencing the genome of the pathogen, the causal agent of High Plains disease was officially named as *High Plains wheat mosaic virus*. Hence, High Plains virus, maize red stripe virus, wheat mosaic virus, and High Plains wheat mosaic virus (HPWMoV) are synonyms for the causal agent of High Plains disease.

**Taxonomy:**

*High Plains wheat mosaic virus* is one of the 21 definitive species in the genus *Emaravirus* in the family *Fimoviridae*.

**Virion:**

The genomic RNAs are encapsidated in thread‐like nucleocapsids in double‐membrane 80–200 nm spherical or ovoid virions.

**Genome characterization:**

The HPWMoV genome consists of eight single‐stranded negative‐sense RNA segments encoding a single open reading frame (ORF) in each genomic RNA segment. RNA 1 is 6,981‐nucleotide (nt) long, coding for a 2,272 amino acid protein of RNA‐dependent RNA polymerase. RNA 2 is 2,211‐nt long and codes for a 667 amino acid glycoprotein precursor. RNA 3 has two variants of 1,439‐ and 1,441‐nt length that code for 286 and 289 amino acid nucleocapsid proteins, respectively. RNA 4 is 1,682‐nt long, coding for a 364 amino acid protein. RNA 5 and RNA 6 are 1,715‐ and 1,752‐nt long, respectively, and code for 478 and 492 amino acid proteins, respectively. RNA 7 and RNA 8 are 1,434‐ and 1,339‐nt long, code for 305 and 176 amino acid proteins, respectively.

**Biological properties:**

HPWMoV can infect wheat, corn (maize), barley, rye brome, oat, rye, green foxtail, yellow foxtail, and foxtail barley. HPWMoV is transmitted by the wheat curl mite and through corn seed.

**Disease management:**

Genetic resistance against HPWMoV in wheat is not available, but most commercial corn hybrids are resistant while sweet corn varieties remain susceptible. Even though corn hybrids are resistant to virus, it still serves as a green bridge host that enables mites to carry the virus from corn to new crop wheat in the autumn. The main management strategy for High Plains disease in wheat relies on the management of green bridge hosts. Cultural practices such as avoiding early planting can be used to avoid mite buildup and virus infections.

## Graphical abstract

HPWMoV, an octapartite single‐stranded RNA virus of negative polarity, is the causal agent of High Plains disease of wheat and corn belongs to the genus *Emaravirus* in the family *Fimoviridae*.

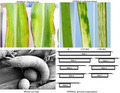

## 1 INTRODUCTION

**FIGURE 1:**
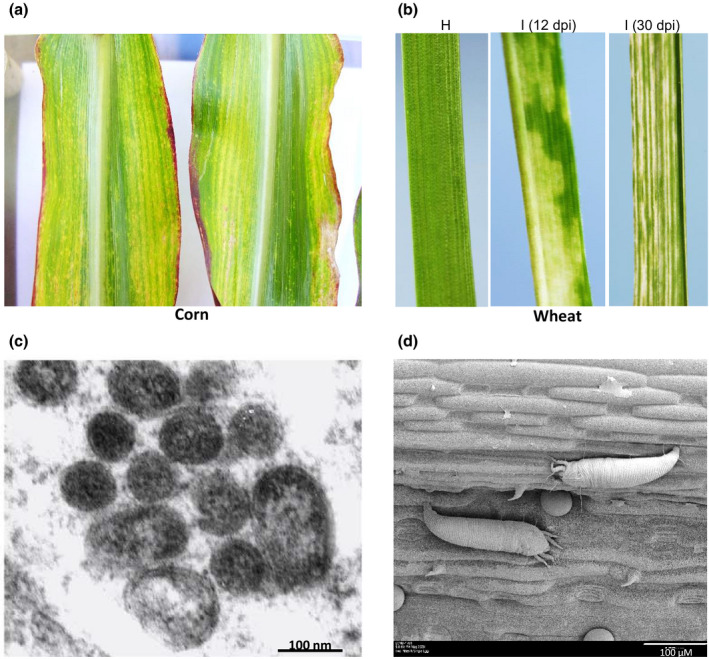
Symptoms induced by High Plains wheat mosaic virus (HPWMoV) on corn leaves under field conditions (a) and on wheat leaves infested with HPWMoV‐viruliferous mites at 12 and 30 days postinfestation (dpi). H, healthy wheat (b). (c) Transmission electron micrograph of HPWMoV virion particles of 80–200 nm diameter in thin tissue sections of virus‐infected corn tissue (Skare et al., 2006). (d) Scanning electron micrograph of two wheat curl mites feeding on a wheat leaf. Note only two pairs of legs located near the front end of the mite and an egg between the mites

High Plains (HP) disease was first identified in 1993 on wheat (*Triticum aestivum*) and corn (maize, *Zea mays*) with severe mosaic and necrosis symptoms in Texas, Kansas, Colorado, Idaho, Nebraska, and Utah (Jensen et al., 1996). Based on the geographical location of infections, the causal agent of HP disease was initially named High Plains virus (HPV; Jensen et al., 1996). The causal agent was found to be transmitted by the wheat curl mite, *Aceria tosichella* (Seifers et al., 1997). Symptoms described for HP disease include mild to severe mosaic, chlorosis, and necrosis on wheat, and chlorotic streaks and red striping on corn (Figure 1a,b). These symptoms are similar to those described for wheat spot mosaic virus (Slykhuis, 1956) and wheat spot chlorosis (Nault et al., 1970) from wheat and corn. Additionally, these pathogens have been reported to be transmitted by the wheat curl mite and produce spherical double‐membrane virus‐like particles similar to those observed for the HP disease pathogen (Figure 1c; Jensen et al., 1996; Nault & Styer, 1970; Seifers et al., 2002; Slykhuis, 1956). These studies suggest that HP disease has probably been present on wheat and corn since the 1950s or earlier.

The causal agent of HP disease was partially purified, and a 32 kDa nucleoprotein was detected along with double‐membrane virus particles (Figure 1c; Jensen et al., 1996). The presence of the 32 kDa nucleoprotein was used as a diagnostic feature of HPV in subsequent studies. The HP disease was subsequently reported from different states in the USA, Argentina, and Australia (Alemandri et al., 2017; Burrows et al., 2009; Coutts et al., 2014; Seifers et al., 2009; Stewart et al., 2013).

In subsequent characterization of the causal agent of HP disease, it was renamed as maize red stripe virus based on the symptoms elicited on corn (Skare et al., 2006). However, the same authors renamed the causal agent of HP disease as wheat mosaic virus because HP disease was first reported on wheat and is more prevalent on wheat than corn. The advent of next‐generation sequencing (NGS) technology facilitated thorough characterization of the genome of the causal agent of HP disease as an octapartite negative‐sense RNA virus (Tatineni et al., 2014). The International Committee on Taxonomy of Viruses approved the name of the causal agent of HP disease as *High Plains wheat mosaic virus*, a distinct member in the genus *Emaravirus*, family *Fimoviridae*.

High Plains wheat mosaic virus (HPWMoV) is one of the components of the wheat streak mosaic disease (WSMD) complex along with wheat streak mosaic virus (WSMV) and Triticum mosaic virus (TriMV). Because the wheat curl mite transmits all the members of the WSMD complex, wheat coinfected by two or three viruses is common in growers’ fields with exacerbated disease phenotype with occasional plant death (Burrows et al., 2009; Byamukama et al., 2013, 2014; Mahmood et al., 1998). The WSMD complex is one of the most economically important diseases of wheat in the USA. Because HPWMoV can be transmitted via seed, it has added potential for global spread through the international transfer of seed.

## 2 AETIOLOGY OF HIGH PLAINS DISEASE

### 2.1 Characterization of the causal agent of HP disease

The 32 kDa viral nucleoprotein was isolated from partially purified preparations from infected tissue by processing it through Triton‐X treatment, followed by ultracentrifugation through 20% sucrose (Jensen et al., 1996). The presence of the 32 kDa protein in partially purified preparations was used as a diagnostic feature for the causal agent of HP disease. Electron microscopy examination of leaf‐dip preparations of HP disease tissue revealed 80–200 nm double‐membrane virus‐like particles (Figure 1c) containing a thread‐like ribonucleoprotein of 32 kDa encapsidating multiple RNA species (Ahn et al., 1996, 1998; Jensen et al., 1996; Skare et al., 2006). Skare et al. (2006) purified HPWMoV virions from field‐collected HP diseased plants through rate zonal sucrose density gradient centrifugation, followed by caesium sulphate isopycnic gradient centrifugation. Viral RNA extracted from this purified preparation resolved into c.8–9 kb RNA, multiple RNA species with sizes ranging between c.2 and 2.5 kb, and a c.1.4 kb RNA. Partial sequencing of the c.1.4 kb RNA revealed that this RNA encodes a 32 kDa protein. This protein was identified as the nucleocapsid (NC) protein based on amino acid identity to a 32 kDa protein sequence obtained through matrix‐assisted laser desorption ionization‐time of flight mass spectrometry (Seifers et al., 2004; She et al., 2004; Skare et al., 2006).

### 2.2 Genome characterization of HPWMoV

**FIGURE 2:**
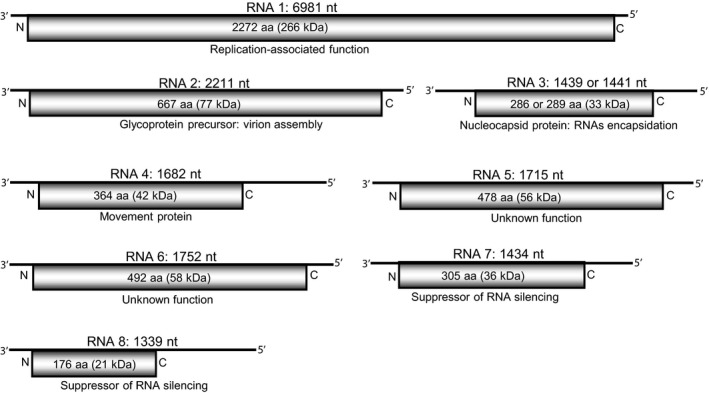
Schematic representation of genome organization of High Plains wheat mosaic virus (HPWMoV). Each schematic diagram represents a genomic RNA segment with an encoded open reading frame (ORF; open rectangles) and 3′‐ and 5′‐nontranslated regions (straight lines). RNA segment number and its size are presented above the genomic organization. The number of amino acids encoded by each ORF and predicted protein size are indicated within the ORF. The function of each protein encoded by eight genomic RNAs is indicated below each ORF. Note that RNA 3A and RNA 3B variants are 1,439 and 1,441 nucleotides long coding for 286 and 289 amino acids, respectively

Despite several attempts of complete genome characterization of the causal agent of HP disease, only a partial or near full‐length sequence was obtained for RNA 3, which contains the sequence complementary to a single open reading frame (ORF) coding for the 32 kDa NC protein (Seifers et al., 2004, 2009; She et al., 2004; Skare et al., 2006). However, the advent of NGS technology revolutionized the genome characterization of recalcitrant viruses (Villamor et al., 2019). The use of virion RNA from partially purified nucleocapsids from HPWMoV‐infected wheat leaves for high‐throughput RNA sequencing facilitated the complete genome sequence of HPWMoV (Tatineni et al., 2014). The genome of HPWMoV consists of eight single‐stranded negative‐sense RNA species encoding a single ORF in each RNA species (Figure 2). Based on the limited sequence homology of RNA segments 1 to 5 with other reported species, HPWMoV was identified as a definitive species of the genus *Emaravirus* (Tatineni et al., 2014). However, RNAs 6 to 8 are unique to HPWMoV with no significant homology with other reported viruses in GenBank. The first 14 nucleotides (nt) of the 5′ end and the last 14 nt of the 3′ end are conserved among all genomic RNA species of HPWMoV. These 14 nt at each end (except for 2 nt) are reverse complementary to each other in all genomic RNA species that form panhandle‐like structures, as observed in the members of other negative‐sense RNA viruses (Falk & Tsai, 1998; Mielke‐Ehret & Mühlbach, 2012; Walter & Barr, 2011).

RNA 1 (6,981 nt) encodes a 266 kDa protein of RNA‐dependent RNA polymerase (P1; RdRp) comprising 2,272 amino acids with 36%–42% sequence identity with other reported emaraviral RdRp proteins (Figure 2). RdRp of HPWMoV contains the following *Bunyaviridae* RdRp signature motifs that are similar to those found in other reported emaraviruses: DXKWS_1114‐1118_ (motif A), QGXXXXXSS_1200‐1208_ (motif B), SDD_1241‐1243_ (motif C), KK_1284‐1285_ (motif D), and EFLST_1294‐1298_ (motif E) (Tatineni et al., 2014). RNA 2 (2,211 nt) encodes a glycoprotein (GP) precursor of 667 amino acis (P2), with a potential cleavage site between Ala_224_ and Asp_225_. The P2 protein possesses 30%–39% amino acid identity with other reported emaraviruses. The predicted cleavage of the GP precursor would release GP1 and GP2 of 25.7 and 50.9 kDa, respectively (Tatineni et al., 2014).

RNA 3 contains two variants, 3A (1,439 nt) and 3B (1,441 nt), with 12.5% sequence divergence (Figure 2; Stewart, 2016; Tatineni et al., 2014). RNA 3A and 3B encode a 33 kDa NC protein (P3) of 286 and 289 amino acids, respectively (Figure 2). Perilla mosaic virus (PerMV) and pistacia virus B (PiVB), tentative species of the genus *Emaravirus*, also contain two variants of RNA 3 with c.17% sequence divergence (Buzkan et al., 2019; Kubota et al., 2020). The significance of two variants of NC proteins in these three emaraviruses is not known. It would be interesting to know how these two NC protein variants encapsidate the genomic RNA species and form double‐membrane virions.

RNA 4 (1,682 nt) encodes a polypeptide of 42 kDa (P4) with 364 amino acids (Figure 2). This protein possesses 46% amino acid identity with the movement protein of raspberry leaf blotch virus (RLBV), suggesting that P4 may be involved in virus movement (Tatineni et al., 2014; Yu et al., 2013). RNA 5 (1,715 nt) of HPWMoV encodes a 478 amino acid polypeptide with a predicted molecular weight of 56 kDa (P5) (Figure 2) with 18%–24% amino acid identities with P5 proteins of fig mosaic virus, RLBV, and pigeonpea sterility mosaic virus‐1 (PPSMV‐1) (Tatineni et al., 2014). RNA 6 (1,752 nt) contains a 492 amino acid ORF with a predicted molecular weight of 58 kDa (P6) (Figure 2). The P6 protein possesses 23% amino acid identity with 72% coverage with RLBV P5 protein (Tatineni et al., 2014). Interestingly, P5 and P6 proteins of HPWMoV possess 26% amino acid identity to each other.

RNA 7 (1,434 nt) and RNA 8 (1,339 nt) encode 36 kDa (P7) and 21 kDa (P8) proteins with 305 and 176 amino acids, respectively (Tatineni et al., 2014). The P7 protein displayed weak amino acid homology with P5 proteins of PerMV, pear chlorotic leaf spot‐associated virus, and Camillia japonica‐associated virus 1 (Kubota et al., 2020; Liu et al., 2020; Zhang et al., 2020). RNA 8 encodes a single ORF comprising only 40% of a 1,339‐nt long RNA segment with an unusually long 715 nt 5′‐nontranslated region (NTR). The P8 protein did not have significant homology with any published GenBank sequences.

**FIGURE 3:**
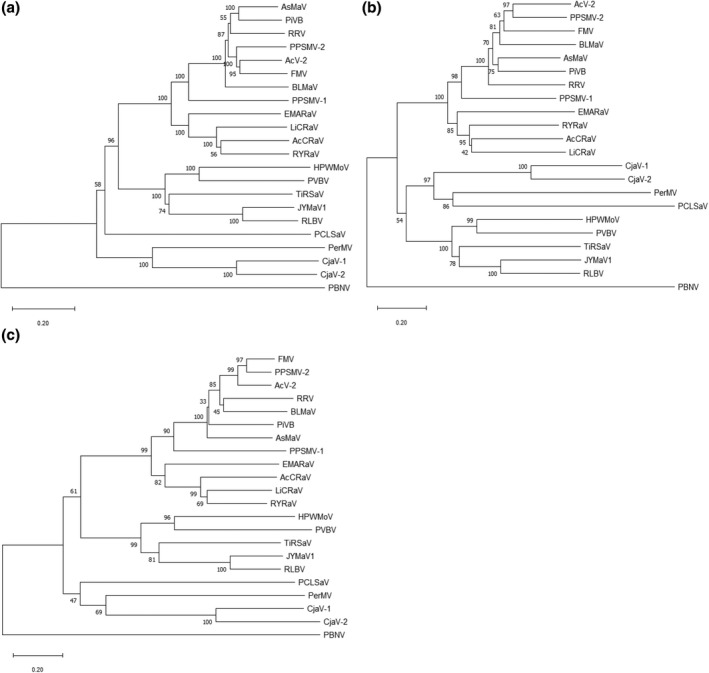
Phylogenetic analyses of definitive members of the genus *Emaravirus* with predicted amino acid sequences of RdRp (a), glycoprotein precursor protein (b), and nucleocapsid protein (c). The corresponding sequences of peanut bud necrosis virus, a tospovirus, were used as an outgroup. The phylogenetic trees were generated with the MEGA v. 11 analysis package (Tamura et al., 2021) with the neighbour‐joining method using the JTT matrix and pairwise gap deletion with 1,000 bootstrap replicates and bootstrap support is indicated at branch points. The bar represents the number of amino acid replacements per site. Note that HPWMoV consistently formed a separate clade with PVBV, TiRSaV, JYMaV1, and RLBV from other members of the genus *Emaravirus*. Virus names, followed by their abbreviations and GenBank accession numbers of RdRp, glycoprotein precursor protein, and nucleocapsid protein, respectively, were presented in the parenthesis: Actinidia chlorotic ringspot‐associated virus (AcCRAV; NC_038769, NC_038770, and NC_0387772); Actinidia emaravirus 2 (AcV‐2; MK602171, MK602172, and MK602173); aspen mosaic‐associated virus (AsMaV; LR742461, LR742462, and LR742463); blackberry leaf mottle associated virus (BLMaV; KY056657, KY056658, and KY056659); Camellia japonica associated emaravirus 1 (CjaV‐1; MN385573, MN385574, and MN385575); Camellia japonica associated emaravirus 2 (CjaV‐2; MN385577, MN385578, and MN385579); European mountain ash ringspot‐associated virus (EMARaV; NC_013105, NC_013106, and NC_013108); fig mosaic virus (FMV; NC_029562, NC_029565, and NC_029563); High Plains wheat mosaic virus (HPWMoV; NC_029570, NC_029549, and NC_029550); jujube yellow mottle‐associated virus (JYMaV1; MK305894, MK305895, and MK305896); lilac chlorotic ringspot‐associated virus (LiCRaV; MT112174, MT112175, and MT112176); Palo verde broom virus (PVBV; MF766025, MF766030, and MF766035); pear chlorotic leaf spot‐associated virus (PCLSaV; MK602177, MK602178, and MK602179); Perilla mosaic virus (PerMV; LC496090, LC496091, and LC496092); pigeonpea sterility mosaic virus 1 (PPSMV‐1; HF568801, HF568802, and HF568803); pigeonpea sterility mosaic virus 2 (PPSMV‐2; NC_030660, NC_030662, and NC_030661); Pistacia emaravirus B (PiVB; MH727572, MH727573, and MH727574); raspberry leaf blotch virus (RLBV; NC_029567, NC_029558, and NC_029559); redbud yellow ringspot virus (RYRaV; NC_038852, NC_038856, and NC_038854); rose rosette virus (RRV; NC_015298, NC_015299, and NC_015300); Ti ringspot‐associated emaravirus (TiRSaV; MH223635, MH223636, and MH223637); and peanut bud necrosis virus (PBNV; NC_003614, NC_003620, and NC_003619)

Phylogenetic analysis of RdRp, glycoprotein precursor, and nucleocapsid proteins of 21 definitive species of emaraviruses resulted in similar topologies by clustering into three distinct clades, suggesting that emaraviruses evolved into three distinct lineages (Figure 3). HPWMoV and Palo verde broom virus (PVBV) formed as sister taxa in a clade along with Ti ringspot‐associated virus (TiRSaV), jujube yellow mottle‐associated virus (JYMaV1), and RLBV. Additionally, HPWMoV and PVBV share a most recent common ancestor with TiRSaV, JYMaV1, and RLBV (Figure 3).

## 3 GENE FUNCTIONS

The P1 protein of HPWMoV is probably involved in viral replication because this protein possesses conserved motifs involved in replication‐associated function. The P2 protein encodes GP precursor protein, which will cleave into GP1 and GP2 at a predicted cleavage site between amino acids 224 and 225. In analogy with distantly related members of bunyaviruses, the glycoproteins of HPWMoV will probably decorate the surface of the double‐membrane virus particle. Because the GP of tospoviruses has been reported to be involved in vector transmission (Sin et al., 2005; Whitfield et al., 2008), the GP of emaraviruses may also be involved in mite transmission. However, experimental evidence is lacking for the role of the GP protein of HPWMoV in wheat curl mite transmission. The NC protein encoded by RNA 3 of HPWMoV protects viral genomic RNAs by forming nucleocapsids, but the significance of two variants of RNA 3 in HPWMoV biology is not known. The P4 protein of HPWMoV has sequence homology with the corresponding proteins of other emaravirus species, particularly with the P4 protein of RLBV. The P4 of RLBV was localized to plasmodesmata and identified as a movement protein (Yu et al., 2013). Based on 46% amino acid identity with the P4 protein of RLBV, the P4 protein of HPWMoV could also be involved in the viral movement. The functions of P5 and P6 proteins in virus biology are not known.

The P7 and P8 proteins of HPWMoV have been reported as suppressors of RNA silencing, and these proteins employ distinct mechanisms of RNA silencing suppression (Gupta et al., 2018, 2019). The P7 and P8 proteins suppressed RNA silencing elicited by single‐ or double‐stranded RNAs and efficiently suppressed the transitive pathway of RNA silencing (Gupta et al., 2018). Moreover, the suppressors of RNA silencing proteins of HPWMoV independently rescued RNA silencing suppressor‐deficient WSMV, a member of the *Potyviridae*, and enhanced the pathogenicity of potato virus X in *Nicotiana benthamiana*. Gupta et al. (2019) found that P7, but not P8, protected long double‐stranded (ds) RNAs from dicing into small interfering RNAs (siRNAs) of 21–24 nt. However, neither protein is bound to long single‐stranded RNAs. The P7 protein bound weakly to 21‐ and 24‐nt ds‐siRNAs, while P8 protein bound strongly to 21‐nt and weakly to 24‐nt ds‐siRNAs, suggesting size‐specific binding. Mutational analysis of conserved GW motif in P7 resulted in loss of RNA silencing suppression activity and pathogenicity. The weak silencing suppression activity of HPWMoV P7 and P8 at the cellular level (Gupta et al., 2018) suggests that the use of a green fluorescent protein (GFP)‐based conventional assay to screen silencing suppression proteins may not be sensitive enough to find weak suppressors of RNA silencing proteins potentially encoded in emaraviruses. Perhaps this could be the reason why there are no reports on RNA silencing suppressor proteins in other emaraviruses despite a large number of new emaravirus species reported during the last 10 years.

## 4 GENE EXPRESSION

Tatineni et al. (2014) found virus‐sense and virus‐complementary (vc)‐sense genomic RNA copies in HPWMoV‐infected wheat tissue at a 10 to 20:1 ratio. Additionally, shorter‐than‐genome length RNAs of vc sense were detected for genomic RNAs 3, 4, 7, and 8, suggesting that these shorter‐than‐genome length RNAs represent subgenomic mRNAs for the expression of ORFs. However, these subgenomic‐length mRNAs were not detected for the genomic RNAs 1, 2, 5, and 6, most probably due to the small size difference between genomic and subgenomic RNAs due to shorter 5′‐NTRs. These data suggest that each genomic RNA of HPWMoV produces genomic‐length virus‐ and vc‐sense RNAs and subgenomic mRNAs of vc sense for gene expression.

## 5 GENETIC VARIABILITY

Since the first report of HPWMoV by Jensen et al. (1996), the NC gene or protein sequence has been reported for several isolates with differential symptoms and infection efficiencies on corn (Seifers et al., 2004, 2009; She et al., 2004; Skare et al., 2006). Seifers et al. (2009) reported two serologically distinct variants, U04‐82 and U04‐83, of HPWMoV from wheat with 32 and 30 kDa NC proteins, respectively, from Kansas. The NC proteins of U04‐82 and U04‐83 isolates, respectively, possessed amino acid identity of 99.6% and 85.5% with isolate ABC5822, and 57% and 50% with isolate TX96 (Seifers et al., 2009), suggesting a high degree of NC protein sequence variability among HPWMoV isolates. The availability of a complete genome sequence of several isolates of HPWMoV facilitated the study of variability in eight genomic RNA segments (Stewart, 2016; Tatineni et al., 2014).

Stewart (2016) reported a near‐complete sequence of a corn isolate from Ohia (isolate GG1) and barley isolate from Kansas (isolate KS7), and a partial sequence of three wheat isolates from Ohio (isolates H1, K1, and W1). The GG1 and KS7 isolates exhibited >98% nucleotide homology with a Nebraska (NE) isolate, while the three Ohio wheat isolates showed <84% sequence homology. The P1 proteins of GG1, KS7, and W1 isolates exhibited 96.2%–99.8% sequence identity with that of the NE isolate. The P2 proteins of isolates GG1 and KS7 showed >99% amino acid identity to the NE isolate, while isolate W1 possessed 92.7% amino acid identity with several nucleotide insertions and deletions in the 5′‐ and 3′‐NTRs compared to a NE isolate (Stewart, 2016).

The RNA 3 sequence of HPWMoV isolates is highly variable, with some isolates possessing two variants and some with only one. HPWMoV isolates NE, GG1, and KS7 have been reported to encode two RNA 3 variants, while wheat isolates from Ohio, isolate ABC58222 from Texas, and isolate U04‐82 from Kansas encode a single RNA 3 species. In phylogenetic analysis, these isolates clustered together in a separate branch from isolates with two variants of RNA 3 (Seifers et al., 2009; Skare et al., 2003; Stewart, 2016; Tatineni et al., 2014). The P3‐A and P3‐B proteins of HPWMoV isolates share 88%–89% sequence identity, and these proteins possess 81%–89% sequence identity with P3 proteins of HPWMoV isolates harbouring a single RNA 3 (Stewart, 2016). It is not clear why some isolates encode two variants of RNA 3 and some isolates encode only a single RNA 3. Seifers et al. (2009) reported isolates U04‐82 and U04‐83 with 32 and 30 kDa NC proteins that showed 86% and 57% identity, respectively, with that of isolate TX96 (Seifers et al., 2009). These data suggest that the NC protein sequence among HPWMoV isolates is highly variable, and NC‐based serological detection of HPWMoV is not a reliable diagnostic method.

The P4 sequence of isolates GG1 and KS7 displayed a high degree of sequence homology (97%–99% amino acid identity) with that of NE isolate with seven indels in the 5′‐NTR (Stewart, 2016). The P5, P6, P7, and P8 proteins of NE, GG1, and KS7 isolates displayed 99%–100% identity, while the Ohio wheat isolates possess 82% (P5), 82%–90% (P6), and 74% (P8) identity with the corresponding proteins of the NE isolate (Stewart, 2016). Sequence comparisons between the isolates revealed that HPWMoV isolates clustered into a group with two RNA 3 variants, such as isolates NE, GG1, and KS7, and a second group with a single RNA 3 including Ohio wheat isolates H1, K1, and W1 (Stewart, 2016). The intragroup sequence comparison of P1 to P8 showed 95%–100% identity and intergroup sequence homology with 92%–93% (P1 and P2), 81%–90% (P3 to P6), and 74% (P8).

## 6 VIRUS TRANSMISSION

### 6.1 Wheat curl mite transmission

HPWMoV is transmitted by the wheat curl mite (Seifers et al., 1997). Wheat curl mites are very small (c.250 µm) and depend on the wind for their dispersal within and between the fields. In eastern Europe and Turkey, the mite has been found to be a species complex of several genetically distinct genotypes (Szydło et al., 2015). Three of these genotypes have been identified in other regions of the world (Skoracka et al., 2014), but only two of these have been found in North America (Hein et al., 2012) and in Australia (Carew et al., 2009). These two genotypes, referred to as type 1 and type 2, have distinct properties related to WSMV transmission (Wosula et al., 2016), host response (Harvey et al., 2001), and survival on various sources of mite resistance genes in wheat (Harvey et al., 1999). Therefore, it is important to understand the differential dynamics of these two mite types in their interactions with their hosts and viruses in this wheat–mite–virus complex.

Vector transmission of HPWMoV isolates with wheat curl mites collected from Kansas, Montana, Nebraska, South Dakota, and Texas found that the Nebraska wheat curl mite population efficiently transmitted HPWMoV. However, the Montana wheat curl mite population transmitted HPWMoV only if the source plants were coinfected with WSMV (Seifers et al., 2002). Hein et al. (2012) found these same five populations used by Seifers et al. (2002) separated into two genetically distinct mite types of wheat curl mites: type 1 (“Kansas” [KS], “Montana” [MT], “South Dakota” [SD], and “Texas” [TX]) and type 2 (“Nebraska” [NE]). Similarly, type 2 mites were found to be efficient transmitters of TriMV, but the type 1 wheat curl mite population failed to transmit TriMV using single mite transfers (McMechan et al., 2014). For WSMV, both mite types can transmit the virus, but type 2 mites were found to be only moderately more effective vectors (Wosula et al., 2016). These data suggest that genetic differences in wheat curl mite play a crucial role in virus–vector interaction for efficient vector transmission.

### 6.2 Mechanical transmission

HPWMoV failed to be transmitted mechanically by rub inoculation to wheat and corn with crude sap from infected leaf tissue. However, HPWMoV isolates could be transmitted to corn by using a vascular puncture inoculation method (Louie et al., 2006; Seifers et al., 2004). In this method, crude sap from infected tissue was inoculated with repeated puncturing along the edge of the seed embryo by a tiny pin. In contrast, a few emaraviruses were mechanically transmitted through crude sap: RLBV and Actinidia chlorotic ring spot‐associated virus onto *Nicotiana benthamiana* (McGavin et al., 2012; Zheng et al., 2017), PPSMV‐1 and PPSMV‐2 onto *N*. *benthamiana* and *Nicotiana clevelandii* (Kumar et al., 2017; Patil & Kumar, 2015), TiRSaV onto *N*. *benthamiana* and *N*. *tabacum* (Olmedo‐Velarde et al., 2019), and rose rosette virus (RRV) onto rose plants (Verchot et al., 2020).

### 6.3 Seed transmission

HPWMoV is transmitted through seed at a very low frequency in sweet corn (Forster et al., 2001). Of 38,473 seedlings grown from HPWMoV‐infected sweet corn seeds, only three seedlings tested positive for HPWMoV. In contrast, in a sweet corn commercial field in 2016 and 2017 in Utah, imaging with an unmanned aerial vehicle with a near‐infrared camera revealed that 2% of the corn plants displayed HP disease‐like symptoms, and they were scattered randomly across the field, a signature feature of a seed‐transmitted pathogen (Nischwitz, 2020). All five symptomatic plants tested from these fields were positive for HPWMoV in ELISA. A partial sequence of these five samples revealed 100% sequence identity with isolate GG1 from corn and isolate KS7 from barley. The remnant sweet corn seed that was used during the 2016 and 2017 seasons obtained from the grower was tested and 70% and 20%, respectively, found positive for HPWMoV by ELISA (Nischwitz, 2020). The lower positivity rate in 2017 sweet corn seed corresponded with fewer symptomatic plants in the field in 2017. In 2016, seed transmission tests under greenhouse conditions indicated six out of 179 seedlings elicited HP disease‐like symptoms, and all six symptomatic plants tested positive for HPWMoV in ELISA and reverse transcription (RT)‐PCR, followed by sequencing (Nischwitz, 2020). These data revealed that HPWMoV is transmitted through seed at a higher percentage than previously reported. These findings confirmed anecdotal reports of the seed transmission nature of HPWMoV at higher rates. Further research is needed to determine the frequency and mechanism of seed transmission of HPWMoV in corn and wheat. Implementing seed testing for the presence of HPWMoV is warranted to prevent the introduction of HPWMoV to other countries through seed import.

## 7 HOST RANGE

HPWMoV was originally reported as infecting wheat and corn (Jensen et al., 1996). The failure to transmit HPWMoV mechanically through rub inoculation made it extremely difficult to study the host range of HPWMoV. Seifers et al. (1998) examined a partial host range of HPWMoV through wheat curl mite transmission and found that HPWMoV can infect wheat, corn, barley (*Hordeum vulgare*), rye brome (*Bromus secalinus*), oat (*Avena sativa*), and rye (*Secale cereale*). Recently, Abdullah et al. (2020) reported that HPWMoV also infects yellow foxtail (*Setaria glauca*) and foxtail barley (*Hordeum jubatum*) plants in Canada.

## 8 VIRUS DIAGNOSIS

In the early days of HP disease discovery, mini‐purification of partially purified virions, followed by sodium dodecyl sulphate‐polyacrylamide gel electrophoresis for the presence of a 32‐kDa nucleocapsid protein was used as a diagnostic method for HPWMoV (Jensen et al., 1996; Seifers et al., 2002). Subsequently, HPWMoV was detected by ELISA (Seifers et al., 1997) and RT‐PCR and quantitative RT‐PCR (RT‐qPCR)‐based molecular diagnostic methods (Arif et al., 2013; Bryan et al., 2019; Elbeaino et al., 2013; Lebas et al., 2005). Arif et al. (2013) modified the primers by adding a customized 22‐nt tail at the 5′ end to increase the melting temperatures of primers. These primers were used for sensitive detection of HPWMoV by SYBR Green and TaqMan RT‐qPCR, endpoint RT‐PCR, RT‐helicase‐dependent amplification, and the Razor Ex BioDetection system. All these methods detected HPWMoV in as little as 1 fg in plasmid DNA carrying the target gene sequence or in infected plant samples. Bryan et al. (2019) developed a RT‐qPCR method for sensitive detection of HPWMoV based on the RNA 3 sequence. This method detected HPWMoV in an additional 17% of ELISA‐negative samples, indicating more sensitive detection of HPWMoV by RT‐qPCR.

## 9 DISEASE CYCLE AND MANAGEMENT STRATEGIES

HPWMoV is transmitted horizontally by the wheat curl mite (Seifers et al., 1997) and vertically through seed (Forster et al., 2001; Nischwitz, 2020). These transmissions play a critical role in completing the HP disease cycle in growers’ fields.

### 9.1 Disease cycle

The epidemiology and disease cycle of all viruses in the wheat–mite–virus complex depend on wheat curl mite ecology and behaviour. Therefore, the viruses most often occur as mixed infections (Burrows et al., 2009; Byamukama et al., 2013). Epidemiological studies of WSMV, the most prominent and extensively studied virus, are also applicable to other eriophyid mite‐transmitted viral diseases. The successful completion of the disease cycle of these viral diseases will depend on the presence of growing plant hosts throughout the year as the mites are unable to survive more than a few days off living host plants (Wosula et al., 2015).

**FIGURE 4:**
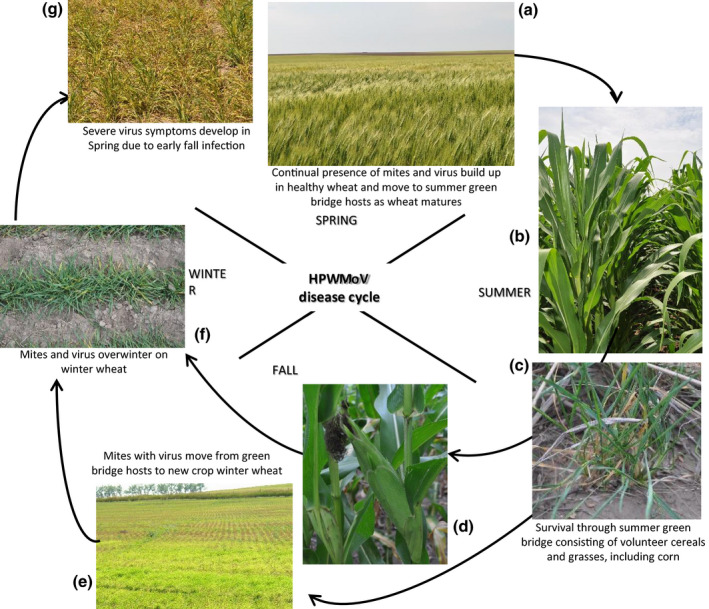
High Plains wheat mosaic virus (HPWMoV) disease cycle in winter wheat–corn cropping systems. Wheat curl mites with virus build up in maturing wheat (a) and move onto summer green bridge grass hosts, including mid‐season corn (b) and especially volunteer wheat resulting from preharvest hail (c). Mites and virus build up within green bridge hosts: symptomless carrier corn (d) and volunteer wheat (c). Mites with virus move from green bridge hosts (volunteer wheat, corn) onto newly planted winter wheat (e) and transmit the virus, and mites and virus overwinter on winter wheat (f). As temperatures warm in the spring, mites become active and can spread the virus, but the most severe virus symptoms that develop and impact wheat (g) result from autumn infections

A major bottleneck in the cycle of this disease complex is survival through the summer, between wheat harvest and emergence of the new wheat crop in the autumn (Figure 4). For the disease cycle to continue, the mites and virus must survive through the summer green bridge period on hosts that are adequate for both to survive (Figure 4). As wheat approaches maturity, mite populations will be very high, and green bridge hosts growing at this time have a high probability of becoming infested by mites (McMechan & Hein, 2017). These high mite populations, along with virus presence, are prevalent in otherwise healthy wheat crops (Byamukama et al., 2016). There are several grass hosts, including corn, that can serve as green bridge hosts for both the mites and viruses. However, the risk of virus spread from most of these hosts is reduced due to poor mite reproduction or host densities too low for significant virus spread (McMechan, 2016). In the central Great Plains of North America, the most problematic green bridge host is volunteer wheat that has emerged before wheat harvest due to hailstorms that shatter grains to the ground. These seeds quickly germinate to produce volunteer wheat that is readily infested by mites moving from the maturing wheat. After wheat harvest, mite activity will be very low, but postharvest emerging volunteer wheat can also become infested, but at very low levels (McMechan, 2016; Staples & Allington, 1956). The longer this postharvest volunteer wheat survives, the greater the chance for significant mite and virus buildup.

An important component of HPWMoV epidemiology is its relationship with corn. Even though most commercial field corn hybrids are resistant to HPWMoV, they do act as symptomless carriers of the virus. Knoell (2018) demonstrated that during the end of the wheat‐growing season, wheat curl mite populations carrying WSMV or HPWMoV move onto corn and reproduce through multiple generations until corn maturity. At the end of the corn‐growing season, mites move onto fall wheat, transmitting both viruses onto wheat. This characteristic of corn being a symptomless carrier of TriMV could not be shown (Knoell, 2018).

In the autumn, wheat curl mites move from the green bridge plants onto newly emerged winter wheat seedlings and transmit HPWMoV. Extended warm temperatures in the autumn provide increased opportunity for mite and virus spread and buildup in the new crop wheat, thus increasing the probability of developing a serious virus epidemic. In winter wheat‐growing areas, wheat curl mites overwinter in winter wheat as eggs, larvae, nymphs, and adult mites, and viruses survive in living plant leaves and crowns (Figure 4). Mites and virus may also survive the winter on some perennial and winter annual grass hosts, but most of these are marginal hosts for the mite. Therefore, winter wheat is the primary host for this disease complex for much of the year because it maintains the mite and virus presence through the winter.

As temperature warms up in the spring, wheat curl mites can again build up and spread the virus (Figure 4), but virus impact in winter wheat from spring‐initiated infections is much less than from autumn infections (Hunger et al., 1992; Wosula et al., 2018). Mite infestations of spring wheat are possible from heavily mite‐infested winter wheat or overwintering volunteer wheat. The earlier spring wheat is infested and infected with virus, the greater the impact from the virus.

### 9.2 Genetic resistance

Genetic resistance in wheat against HPWMoV is not known. However, wheat cultivars with nonallelic *Wsm1*, *Wsm2*, and *Wsm3* genes provide resistance against WSMV, but only *Wsm1* and *Wsm3* also provide resistance against TriMV (Danilova et al., 2017; Graybosch et al., 2009; Lu et al., 2011). Only the resistance of *Wsm3* against WSMV appears to be stable at increased temperatures (Danilova et al., 2017). However, the level of protection these genes provide against HPWMoV is not clear. Genetic markers in corn inbred lines for resistance against HPWMoV and WSMV cosegregate (see below), therefore it is plausible that wheat cultivars with *Wsm1*, *Wsm2*, and *Wsm3* genes that confer resistance to WSMV may also provide a similar kind of resistance to HPWMoV. However, screening of wheat cultivars with these WSMV‐resistance genes needs to be performed against HPWMoV.

Because HP disease was first observed with more symptom severity in corn compared to wheat, Marçon et al. (1997a) screened several inbred corn lines for resistance against HPWMoV. Corn inbred lines B73 and B14, and lines W64A, Wf9, H100, N213, N215, and N194 were found resistant and susceptible, respectively, to HPWMoV. The corn inbred line B73 was also found to be resistant to WSMV. Resistance to HPWMoV in B73 was mapped by crossing to susceptible corn lines Wf9 and W64A to reveal that all F_1_ plants were resistant to HPWMoV (Marçon et al., 1997b). F_2_ plants segregated at a 3:1 (resistant:susceptible) ratio, indicating a single dominant gene is responsible for resistance in B73. The resistance allele in B73 was linked to marker *bn16.29* on the short arm of chromosome 6.

Marçon et al. (1999) characterized the inheritance of resistance of B73 to HPWMoV and WSMV by crossing resistant B73 with Mo17, a moderately susceptible line. They mapped major resistance genes for systemic infection by HPWMoV to chromosomes 3 and 6, and the resistance genes are tightly linked to the WSMV resistance loci. Genetic analyses of inbred corn lines revealed that the majority of lines are resistant to both HPWMoV and WSMV, and loci conferring resistance to both viruses on chromosomes 3, 6, and 10 are consistent with the map position of *Wsm1*, *Wsm2*, and *Wsm3* genes, respectively (Marçon et al., 1999). When HPWMoV was detected in the Great Plains in 1993 and 1994, it was seen in only a few susceptible lines, and these susceptible hybrids were eliminated quickly such that commercial hybrids grown throughout the Great Plains now show resistance to HPWMoV. However, sweet corn hybrids are quite susceptible to HPWMoV, particularly when infected at early growth stages (Revilla et al., 2021).

Because HPWMoV is transmitted through wheat curl mites, the development of crop plants resistant to wheat curl mites could provide an effective management strategy not only to HP disease but also against WSMV and TriMV. Additionally, the development of mite‐resistant crop plants could prevent or minimize losses incurred due to wheat curl mite feeding. Thus, mite‐resistant crop plants will provide dual protection from viruses as well as feeding damage by wheat curl mites. So far, in wheat, four curl mite colonization (*Cmc*) genes, *Cmc1*/*Cmc4*, *Cmc2*, or *Cmc3*, with DNA transferred from *Aegilops tauschii*, *Agropyron elongatum*, and *S*. *cereale*, respectively, have been reported to provide genetic resistance against wheat curl mite (Thomas et al., 2004). The extensive deployment of the *Cmc3* gene in wheat (cv. TAM107) resulted in adaptation of resistance by wheat curl mite populations to the *Cmc* gene, enabling them to readily colonize the wheat (Harvey et al., 1997). Thus, the stability of these cultivars’ resistance against wheat curl mites is uncertain. However, developing wheat cultivars with gene pyramiding of multiple wheat curl mite resistant genes with virus‐resistant genes may help stabilize resistance against this mite–virus complex.

### 9.3 Cultural practices

Control of wheat curl mites through pesticide application for management of HP disease is ineffective due to the secluded nature of mites within the whorl of wheat plants. Because HPWMoV most often occurs in mixed infections in wheat (Burrows et al., 2009; Byamukama et al., 2013), cultural practices developed for other wheat curl mite‐transmitted viruses such as WSMV in wheat are applicable for managing the HP disease. Among cultural practices, interruption of the disease cycle by eliminating high‐risk volunteer wheat for approximately 2 weeks during this green bridge period will minimize the risk of significant virus spread for the next growing season (Wegulo et al., 2008).

For winter wheat, avoiding early planting is recommended to reduce the overlap of green bridge hosts and the new crop wheat. This reduces the chances of direct infestation from the green bridge host, but it also reduces mite and virus buildup and spread during favourable warm autumn temperatures. The effective management of HP disease should include the integration of cultural practices and genetic resistance. Thus, considerably more needs to be learned about HPWMoV resistance relationships in developing wheat lines with WSMV resistance.

In areas where winter wheat is grown in cropping systems that include corn, management of HPWMoV in commercial corn is largely accomplished through virus resistance in corn hybrids. This eliminates the impact of the virus on corn production. Corn can still serve as a green bridge host to carry mites and virus to surrounding wheat fields (Knoell, 2018), so it is important to minimize the overlap of winter wheat emergence in the autumn and late maturing corn. If winter wheat is in a cropping system with sweet corn, management of HPWMoV will require avoiding planting dates that allow the early vegetative stage of corn during the later stages of wheat maturity when mite movement from wheat is greatest.

## 10 CONCLUSION AND FUTURE DIRECTIONS

The causal agent of HP disease was determined to be an octapartite negative‐sense RNA virus, and it was named *High Plains wheat mosaic virus* in the genus *Emaravirus*, family *Fimoviridae* (Tatineni et al., 2014). The use of virion RNA isolated from partially purified nucleocapsids for high‐throughput RNA sequencing facilitated identifying eight genomic RNA species in the genome of HPWMoV. This is the first report of a member of the genus *Emaravirus* that contains eight genomic RNA segments. Subsequent sequencing of several isolates from wheat, corn, and barley from Kansas and Ohio revealed sequence diversity with two RNA 3 segments in isolates GG1 and KS7 but only one RNA 3 segment in Ohio wheat isolates (Stewart, 2016). The sequences of several isolates of HPWMoV from different host species from different locations in the USA and around the world are needed to provide a more complete picture of the diversity in the isolates of HPWMoV.

The next step after sequencing the HPWMoV genomic RNAs is determining the functions of virus‐encoded proteins. Based on sequence similarity, the genes encoded by RNA 1, 2, 3, and 4 of HPWMoV were identified as replication‐associated proteins, glycoprotein‐precursor, nucleocapsid, and movement protein, respectively (Tatineni et al., 2014). The proteins encoded by RNA 7 and 8 were identified as suppressors of RNA silencing with distinct, nonoverlapping mechanisms to counter the host defence mechanisms (Gupta et al., 2018, 2019). The function of proteins encoded by RNA 5 and 6 is not known. It is possible that some of the HPWMoV‐encoded proteins may have multiple functions in virus biology. A reverse genetics system could be used to determine if all the RNAs are actually needed for infection. For example, are both 3A and 3B needed for encapsidation of genomic RNAs and what virus‐encoded proteins are required for wheat curl mite transmission and disease development? A binary vector‐based reverse genetics system was recently developed for a negative‐sense RNA virus, RRV, an *Emaravirus* (Verchot et al., 2020). This study may lead to the development of a similar reverse genetics system for HPWMoV. Studies on replication, transcription and gene expression, virus movement, and virion assembly will facilitate the examination of virus biology. These studies could provide important clues to the functioning of other negative‐strand RNA viruses infecting plants, animals, and humans.

The development of sensitive and broad‐spectrum detection methods will facilitate the detection of HPWMoV isolates in alternate hosts. Though PCR‐based methods were reported (Arif et al., 2013; Bryan et al., 2019; Elbeaino et al., 2013; Lebas et al., 2005) for HPWMoV detection, ELISA‐based detection would provide a simpler large‐scale detection method. Sequence diversity in the nucleocapsid gene precludes the use of currently available ELISA‐based detection methods for efficient broad‐scale detection of HPWMoV isolates. However, large‐scale sequencing of the nucleocapsid gene of HPWMoV from different isolates could provide a conserved peptide sequence that could be used for polyclonal or monoclonal antibodies for ELISA‐based detection methods.

Viruses are obligate parasites, hence they must depend on host and vector machinery for replication, movement, disease development, and transmission. Examination of virus–host interactions will facilitate the identification of host factors that can be used as targets to disrupt the virus life cycle by targeting host proteins. Identification of host factors that do not have a crucial role in plant growth and development but are required for virus life cycle could be candidates for use in the management of viral diseases. The mechanisms of wheat curl mite transmission of HPWMoV are not known. It is not known whether wheat curl mites transmit the virus in a persistent, nonpersistent, or semipersistent manner or if the virus replicates within the mite. Virus–vector interaction studies will facilitate the identification of wheat curl mite proteins required for HPWMoV transmission. These mite proteins could be used as targets to knockout wheat curl mite transmission of HPWMoV. Studies on HPWMoV gene functions and identification of host and vector proteins interacting with HPWMoV proteins could eventually facilitate new management strategies. Candidate host proteins involved in disease development could be targeted via CRISPR/Cas9 technology to interrupt virus–host interactions and mitigate the viruses’ ability to infect plants.

Seed transmission of HPWMoV in corn creates serious issues in sweet corn because of the susceptibility of sweet corn hybrids. This is also an issue in hybrid seed corn production that is targeted for export as seed lots testing positive for HPWMoV are at risk of embargo. Understanding the virus–host relationship that leads to HPWMoV seed transmission could enable the development of technology targeting the interruption of seed transmission.

Corn and wheat cultivars with *Wsm* genes provide resistance against WSMV. However, it is not known whether these cultivars provide similar resistance against HPWMoV. It will be important to examine whether *Wsm*‐based corn and wheat cultivars provide a similar level of resistance to HPWMoV. If any of these genes are also resistant to HPWMoV in wheat, gene pyramiding of these genes with wheat curl mite resistant *Cmc* genes could provide a dual resistance to virus and vector.

## CONFLICTS OF INTEREST

The authors declare no conflict of interest.

## Data Availability

Data sharing is not applicable to this article as no new data were created or generated in this study.

## References

[mpp13113-bib-0001] Abdullah, I., Bennypaul, H., Phelan, J., Aboukhaddour, R. & Harding, M.W. (2020) First report of High Plains wheat mosaic emaravirus infecting foxtail and wheat in Canada. Plant Disease, 104, 3272.

[mpp13113-bib-0002] Ahn, K.K., Kim, K.S., Gergerich, R.C. & Jensen, S.G. (1998) High plains disease of corn and wheat: ultrastructural and serological aspects. Journal of Submicroscopic Cytology and Pathology, 30, 563–571. 9851064

[mpp13113-bib-0003] Ahn, K.K., Kim, K.S., Gergerich, R.C., Jensen, S.G. & Anderson, E.J. (1996) Comparative ultrastructure of double membrane‐bound particles and inclusions associated with eriophyid mite‐borne plant disease of unknown etiology: a potentially new group of plant viruses. Journal of Submicroscopic Cytology and Pathology, 28, 345–355.

[mpp13113-bib-0004] Alemandri, V., Mattio, M.F., Rodriguez, S.M. & Truol, G. (2017) Geographical distribution and first molecular detection of an *Emaravirus*, High Plains wheat mosaic virus, in Argentina. European Journal of Plant Pathology, 149, 743–750.

[mpp13113-bib-0005] Arif, M., Aguilar‐Moreno, G.S., Wayadande, A., Fletcher, J. & Ochoa‐Corona, F.M. (2013) Primer modification improves rapid and sensitive *in vitro* and field deployable assays for detection of High Plains virus variants. Applied and Environmental Microbiology, 80, 320–327. 24162574 10.1128/AEM.02340-13 PMC3910988

[mpp13113-bib-0006] Bryan, B., Paetzold, L., Workneh, F. & Rush, C.M. (2019) Incidence of mite‐vectored viruses of wheat in the Texas high plains and interactions with their host and vector. Plant Disease, 103, 2996–3001. 31560615 10.1094/PDIS-03-19-0620-SR

[mpp13113-bib-0007] Burrows, M., Franc, G., Rush, C., Blunt, T., Ito, D., Kinzer, K. et al. (2009) Occurrence of viruses in wheat in the Great Plains region 2008. Plant Health Progress, 10, 14.

[mpp13113-bib-0008] Buzkan, N., Chiumenti, M., Massart, S., Sarpkaya, K., Karadağ, S. & Minafra, A. (2019) A new Emaravirus discovered in Pistacia from Turkey. Virus Research, 263, 159–163. 30682378 10.1016/j.virusres.2019.01.012

[mpp13113-bib-0009] Byamukama, E., Seifers, D.L., Hein, G.L., De Wolf, E., Tisserat, N.A., Langham, M.A.C. et al. (2013) Occurrence and distribution of *Triticum mosaic virus* in the Central Great Plains. Plant Disease, 97, 21–29. 30722266 10.1094/PDIS-06-12-0535-RE

[mpp13113-bib-0010] Byamukama, E., Tatineni, S., Hein, G.L., McMechan, A.J. & Wegulo, S.N. (2016) Incidence of *Wheat streak mosaic virus*, *Triticum mosaic virus*, and *Wheat mosaic virus* in wheat curl mites recovered from maturing winter wheat spikes. Plant Disease, 100, 318–323. 30694138 10.1094/PDIS-06-15-0692-RE

[mpp13113-bib-0011] Byamukama, E., Wegulo, S., Tatineni, S., Hein, G.L., Graybosch, R., Baenziger, P.S. et al. (2014) Quantification of yield loss caused by *Triticum mosaic virus* and *Wheat streak mosaic virus* in winter wheat under field conditions. Plant Disease, 98, 127–133. 30708611 10.1094/PDIS-04-13-0419-RE

[mpp13113-bib-0012] Carew, M., Schiffer, M., Umina, P., Weeks, A. & Hoffmann, A. (2009) Molecular markers indicate that the wheat curl mite, *Aceria tosichella* Keifer, may represent a species complex in Australia. Bulletin of Entomological Research, 99, 479–486. 19224660 10.1017/S0007485308006512

[mpp13113-bib-0013] Coutts, B.A., Cox, B.A., Thomas, G.J. & Jones, R.A.C. (2014) First report of Wheat mosaic virus infecting wheat in Western Australia. Plant Disease, 98, 285. 10.1094/PDIS-03-13-0288-PDN 30708758

[mpp13113-bib-0014] Danilova, T.V., Zhang, G., Liu, W., Fribe, B. & Gill, B.S. (2017) Homoeologous recombination‐based transfer and molecular cytogenetic mapping of a wheat streak mosaic virus and *Triticum* mosaic virus resistance gene *Wsm3* from *Thinopyrum intermedium* to wheat. Theoretical and Applied Genetics, 130, 549–556. 27900400 10.1007/s00122-016-2834-8

[mpp13113-bib-0015] Elbeaino, T., Whitfield, A., Sharma, M. & Digiaro, M. (2013) Emaravirus‐specific degenerate PCR primers allowed the identification of partial RNA‐dependent RNA polymerase sequences of Maize red stripe virus and Pigeonpea sterility mosaic virus. Journal of Virological Methods, 188, 37–40. 23219928 10.1016/j.jviromet.2012.11.037

[mpp13113-bib-0016] Falk, B. & Tsai, J.H. (1998) Biology and molecular biology of viruses in the genus tenuiviruses. Annual Review of Phytopathology, 36, 139–163. 10.1146/annurev.phyto.36.1.139 15012496

[mpp13113-bib-0017] Forster, R.L., Seifers, D.L., Strausbaugh, C.A., Jensen, S.G., Ball, E.M. & Harvey, T.L. (2001) Seed transmission of the High Plains virus in sweet corn. Plant Disease, 85, 696–699. 30823191 10.1094/PDIS.2001.85.7.696

[mpp13113-bib-0018] Graybosch, R.A., Peterson, C.J., Baenziger, P.S., Baltensperger, D.D., Nelson, L.A., Jin, Y. et al. (2009) Registration of ‘Mace’ hard red winter wheat. Journal of Plant Registrations, 3, 51–56.

[mpp13113-bib-0019] Gupta, A.K., Hein, G.L., Graybosch, R.A. & Tatineni, S. (2018) Octapartite negative‐sense RNA genome of *High Plains wheat mosaic virus* encodes two suppressors of RNA silencing. Virology, 518, 152–162. 29499560 10.1016/j.virol.2018.02.013

[mpp13113-bib-0020] Gupta, A.K., Hein, G.L. & Tatineni, S. (2019) P7 and P8 proteins of *High Plains wheat mosaic virus*, a negative‐strand RNA virus, employ distinct mechanisms of RNA silencing suppression. Virology, 535, 20–31. 31254744 10.1016/j.virol.2019.06.011

[mpp13113-bib-0021] Harvey, T.L., Martin, T.J., Seifers, D.L. & Sloderbeck, P.E. (1997) Change in virulence of wheat curl mite detected on TAM 107 wheat. Crop Science, 37, 624–625.

[mpp13113-bib-0022] Harvey, T.L., Seifers, D.L. & Martin, T.J. (2001) Host range differences between two strains of wheat curl mites (Acari: Eriophyidae). Journal of Agricultural and Urban Entomology, 18, 35–41.

[mpp13113-bib-0023] Harvey, T.L., Seifers, D.L., Martin, T.J., Brown‐Guedira, G. & Gill, B.S. (1999) Survival of wheat curl mites on different sources of resistance in wheat. Crop Science, 39, 1887–1889.

[mpp13113-bib-0024] Hein, G.L., French, R., Siriwetwiwat, B. & Amrine, J.W. (2012) Genetic characterization of North American populations of wheat curl mite and dry bulb mite. Journal of Economic Entomology, 105, 1801–1808. 23156180 10.1603/ec11428

[mpp13113-bib-0025] Hunger, R.M., Sherwood, J.L., Evans, C.K. & Montana, J.R. (1992) Effects of planting date and inoculation date on severity of wheat streak mosaic in hard red winter wheat cultivars. Plant Disease, 76, 1056–1060.

[mpp13113-bib-0026] Jensen, S.G., Lane, L.C. & Seifers, D.L. (1996) A new disease of maize and wheat in the High Plains. Plant Disease, 80, 1387–1390.

[mpp13113-bib-0027] Knoell, E. (2018) Transmission characteristics of Triticum mosaic virus by the wheat curl mite and ecology of the wheat‐mite virus complex on field corn. M.S. Thesis, Lincoln, NE: University of Nebraska‐Lincoln.

[mpp13113-bib-0028] Kubota, K., Usugi, T., Tomitaka, Y., Shimomoto, Y., Takeuchi, S., Kadono, F. et al. (2020) Perilla mosaic virus is a highly divergent Emaravirus transmitted by *Shevtchenkella* sp. (acari: *Eriophyidae*). Phytopathology, 110, 1352–1361. 32202482 10.1094/PHYTO-01-20-0013-R

[mpp13113-bib-0029] Kumar, S., Subbarao, B.L. & Hallan, V. (2017) Molecular characterization of emaraviruses associated with Pigeonpea sterility mosaic disease. Scientific Reports, 7, 11831. 28928453 10.1038/s41598-017-11958-8 PMC5605523

[mpp13113-bib-0030] Lebas, B.S.M., Ochoa‐Corona, F.M., Elliott, D.R., Tang, E.Z. & Alexander, B.J.R. (2005) Development of an RT‐PCR for High Plains virus indexing scheme in New Zealand post‐entry quarantine. Plant Disease, 89, 1103–1108. 30791279 10.1094/PD-89-1103

[mpp13113-bib-0031] Liu, H., Wang, G., Yang, Z., Wang, Y., Zhang, Z., Li, L. et al. (2020) Identification and characterization of a pear chlorotic leaf spot‐associated virus, a novel emaravirus associated with a severe disease of pear trees in China. Plant Disease, 104, 2786–2798. 32997610 10.1094/PDIS-01-20-0040-RE

[mpp13113-bib-0032] Louie, R., Seifers, D.L. & Bradfute, O.E. (2006) Isolation, transmission and purification of the High Plains virus. Journal of Virological Methods, 135, 214–222. 16672165 10.1016/j.jviromet.2006.03.023

[mpp13113-bib-0033] Lu, H., Price, J., Devkota, R., Rush, C. & Rudd, J. (2011) A dominant gene for resistance to wheat streak mosaic virus in winter wheat line CO960293‐2. Crop Science, 51, 5–12.

[mpp13113-bib-0034] Mahmood, T., Hein, G.L. & Jenesen, S.G. (1998) Mixed infection of hard red winter wheat with High Plains virus and Wheat streak mosaic virus from wheat curl mites in Nebraska. Plant Disease, 82, 311–315. 30856864 10.1094/PDIS.1998.82.3.311

[mpp13113-bib-0035] Marçon, A., Kaeppler, S.M. & Jensen, S.G. (1997a) Genetic variability among maize inbred lines for resistance to the High Plains virus‐wheat streak mosaic virus complex. Plant Disease, 81, 195–198. 30870896 10.1094/PDIS.1997.81.2.195

[mpp13113-bib-0036] Marçon, A., Kaeppler, S.M. & Jensen, S.G. (1997b) Resistance to systemic spread of High Plains virus and Wheat streak mosaic virus cosegregates in two F2 maize populations inoculated with both pathogens. Crop Science, 37, 1923–1927.

[mpp13113-bib-0037] Marçon, A., Kaeppler, S.M., Jensen, S.G., Senior, L. & Stuber, C. (1999) Loci controlling resistance to High Plains virus and Wheat streak mosaic virus in a B73 × Mo17 population of maize. Crop Science, 39, 1171–1177.

[mpp13113-bib-0038] McGavin, W.J., Mitchell, C., Cock, P.J.A., Wright, K.M. & MacFarlane, S.A. (2012) Raspberry leaf blotch virus, a putative new member of the genus Emaravirus, encodes a novel genomic RNA. Journal of General Virology, 93, 430–437. 10.1099/vir.0.037937-0 22049090

[mpp13113-bib-0039] McMechan, A.J. (2016) Oversummering (green bridge) ecology of the wheat curl mite (Aceria tosichella Keifer). Ph. D. Dissertation, Lincoln, NE: University of Nebraska–Lincoln.

[mpp13113-bib-0040] McMechan, A.J. & Hein, G.L. (2017) Population dynamics of the wheat curl mite (Acari: Eriophyidae) during the heading stages of winter wheat. Journal of Economic Entomology, 110, 355–361. 28334133 10.1093/jee/tox028

[mpp13113-bib-0041] McMechan, A.J., Tatineni, S., French, R. & Hein, G.L. (2014) Differential transmission of Triticum mosaic virus by wheat curl mite populations collected in the Great Plains. Plant Disease, 98, 806–810. 30708632 10.1094/PDIS-06-13-0582-RE

[mpp13113-bib-0042] Mielke‐Ehret, N. & Mühlbach, H.P. (2012) *Emaravirus*: a novel genus of multipartite, negative strand RNA plant viruses. Viruses, 4, 1515–1536. 23170170 10.3390/v4091515 PMC3499817

[mpp13113-bib-0043] Nault, L.R. & Styer, W.E. (1970) Transmission of an Eriophyid‐borne wheat pathogen by *Aceria tulipae* . Phytopathology, 60, 1616–1618.

[mpp13113-bib-0044] Nault, L.R., Styer, W.E., Gordon, D.T., Bradfute, O.E., Lafever, H.N. & William, L.E. (1970) An eriophyid‐borne pathogen from Ohio, and its relation to wheat spot mosaic virus. Plant Disease Report, 54, 156–160.

[mpp13113-bib-0045] Nischwitz, C. (2020) Seed‐transmitted wheat mosaic virus in Utah. Plant Health Progress, 21, 212–213.

[mpp13113-bib-0046] Olmedo‐Velarde, A., Park, A.C., Sugano, J., Uchida, J.Y., Kawate, M., Borth, W.B. et al. (2019) Characterization of Ti ringspot‐associated virus, a novel Emaravirus associated with an emerging ringspot disease of *Cordyline fruticose* . Plant Disease, 103, 2345–2352. 31306086 10.1094/PDIS-09-18-1513-RE

[mpp13113-bib-0047] Patil, B.L. & Kumar, P.L. (2015) *Pigeonpea sterility mosaic virus*: a legume‐infecting *Emaravirus* from South Asia. Molecular Plant Pathology, 16, 775–786. 25640756 10.1111/mpp.12238 PMC6638375

[mpp13113-bib-0048] Revilla, P., Anibas, C.M. & Tracy, W.F. (2021) Sweet corn research around the world 2015–2020. Agronomy, 11, 534.

[mpp13113-bib-0049] Seifers, D.L., Harvey, T.L., Louie, R., Gordon, D.T. & Martin, T.J. (2002) Differential transmission of isolates of the High Plains virus by different sources of wheat curl mites. Plant Disease, 86, 138–142. 30823310 10.1094/PDIS.2002.86.2.138

[mpp13113-bib-0050] Seifers, D.L., Harvey, T.L., Martin, T.J. & Jensen, S.G. (1997) Identification of the wheat curl mite as the vector of the High Plains virus of corn and wheat. Plant Disease, 81, 1161–1166.

[mpp13113-bib-0051] Seifers, D.L., Harvey, T.L., Martin, T.J. & Jensen, S.G. (1998) A partial host range of the High Plains virus of corn and wheat. Plant Disease, 82, 875–879. 30856913 10.1094/PDIS.1998.82.8.875

[mpp13113-bib-0052] Seifers, D.L., Martin, T.J., Harvey, T.L., Fellers, J.P. & Michaud, J.P. (2009) Identification of the wheat curl mite as the vector of Triticum mosaic virus. Plant Disease, 93, 25–29. 30764256 10.1094/PDIS-93-1-0025

[mpp13113-bib-0053] Seifers, D.L., She, Y.‐M., Harvey, T.L., Martin, T.J., Haber, S., Ens, W. et al. (2004) Biological and molecular variability among High Plains virus isolates. Plant Disease, 88, 824–829. 30812509 10.1094/PDIS.2004.88.8.824

[mpp13113-bib-0054] She, Y.‐M., Seifers, D.L., Haber, S., Ens, W. & Standing, K.G. (2004) Characterization of the agent of ‘High Plains Disease’: mass spectrometry determines the sequence of the disease‐specific protein. Journal of Biological Chemistry, 279, 488–494. 10.1074/jbc.M308506200 14561770

[mpp13113-bib-0055] Sin, S.‐H., McNulty, B.C., Kennedy, G.G. & Moyer, J.W. (2005) Viral genetic determinants for thrips transmission of tomato spotted wilt virus. Proceedings of the National Academy of Sciences of the United States of America, 102, 5168–5173. 15753307 10.1073/pnas.0407354102 PMC552972

[mpp13113-bib-0056] Skare, J.M., Wijkamp, I., Denham, I., Rezende, J.A.M., Kitajima, E.W., Park, J.‐W. et al. (2006) A new eriophyid mite‐borne membrane enveloped virus‐like complex isolated from plants. Virology, 347, 343–353. 16412487 10.1016/j.virol.2005.11.030

[mpp13113-bib-0057] Skare, J.M., Wijkamp, I., Rezende, J., Michels, G., Rush, C., Scholthof, K.‐B.‐G. et al. (2003) Colony establishment and maintenance of the eriophyid wheat curl mite *Aceria tosichella* for controlled transmission studies on a new virus‐like pathogen. Journal of Virological Methods, 108, 133–137. 12565164 10.1016/s0166-0934(02)00257-4

[mpp13113-bib-0058] Skoracka, A., Rector, B., Kuszynski, L., Szydło, W., Hein, G.L. & French, R. (2014) Global spread of wheat curl mite by its most polyphagous and pestiferous lineages. Annals of Applied Biology, 165, 222–235.

[mpp13113-bib-0059] Slykhuis, J.T. (1956) Wheat spot mosaic, caused by a mite‐transmitted virus associated with wheat streak mosaic virus. Phytopathology, 46, 682–687.

[mpp13113-bib-0060] Staples, R. & Allington, W.B. (1956) Streak mosaic of wheat in Nebraska and its control. Research Bulletin of the Nebraska Agricultural Experimental Station, 178.

[mpp13113-bib-0061] Stewart, L.R. (2016) Sequence diversity of wheat mosaic virus isolates. Virus Research, 213, 299–303. 26590326 10.1016/j.virusres.2015.11.013

[mpp13113-bib-0062] Stewart, L.R., Paul, P.A., Qu, F., Redinbaugh, M.G., Miao, H., Todd, J. et al. (2013) Wheat mosaic virus (WMoV), the causal agent of High Plains disease, is present in Ohio wheat fields. Plant Disease, 97, 1125. 10.1094/PDIS-03-13-0243-PDN 30722503

[mpp13113-bib-0063] Szydło, W., Hein, G.L., Denizhan, E. & Skoracka, A. (2015) Exceptionally high levels of genetic diversity in wheat curl mite (Acari: Eriophyidae) populations from Turkey. Journal of Economic Entomology, 108, 2030–2039. 26470350 10.1093/jee/tov180

[mpp13113-bib-0064] Tamura, K., Stecher, G. & Kumar, S. (2021) MEGA11: molecular evolutionary genetics analysis version 11. Molecular Biology and Evolution, 38, 3022–3027. 33892491 10.1093/molbev/msab120 PMC8233496

[mpp13113-bib-0065] Tatineni, S., McMechan, A.J., Wosula, E.N., Wegulo, S.N., Graybosch, R.A., French, R. et al. (2014) An eriophyid mite‐transmitted plant virus contains eight genomic RNA segments with unusual heterogeneity in the nucleocapsid protein. Journal of Virology, 88, 11834–11845. 25100845 10.1128/JVI.01901-14 PMC4178757

[mpp13113-bib-0066] Thomas, J.B., Conner, R.L. & Graf, R.J. (2004) Comparison of different sources of vector resistance for controlling Wheat streak mosaic in winter wheat. Crop Science, 44, 250130.

[mpp13113-bib-0067] Verchot, J., Herath, V., Urrutia, C.D., Gayral, M., Lyle, K., Shires, M.K. et al. (2020) Development of a reverse genetic system for studying rose rosette virus in whole plants. Molecular Plant‐Microbe Interactions, 33, 1209–1221. 32815767 10.1094/MPMI-04-20-0094-R

[mpp13113-bib-0068] Villamor, D.E.V., Ho, T., Al Rwahnih, M., Martin, R.R. & Tzanetakis, I.E. (2019) High throughput sequencing for plant virus detection and discovery. Phytopathology, 109, 716–725. 30801236 10.1094/PHYTO-07-18-0257-RVW

[mpp13113-bib-0069] Walter, C.T. & Barr, J.N. (2011) Recent advances in the molecular and cellular biology of bunyaviruses. Journal of General Virology, 92, 2467–2484. 10.1099/vir.0.035105-0 21865443

[mpp13113-bib-0070] Wegulo, S.N., Hein, G.L., Klein, N. & French, R.C. (2008) Managing wheat streak mosaic. Univ. Neb.‐Linc. Ext. EC1871.

[mpp13113-bib-0071] Whitfield, A.E., Kumar, N.K.K., Rotenberg, D., Ullman, D.E., Wyman, E.A., Zietlow, C. et al. (2008) A soluble form of the Tomato spotted wilt virus (TSWV) glycoprotein GN (GN‐S) inhibits transmission of TSWV by *Frankliniella occidentalis* . Phytopathology, 98, 45–50. 18943237 10.1094/PHYTO-98-1-0045

[mpp13113-bib-0072] Wosula, E.N., McMechan, A.J. & Hein, G.L. (2015) The effect of temperature, relative humidity, and virus infection status on off‐host survival of the wheat curl mite (Acari: Eriophyidae). Journal of Economic Entomology, 108, 1545–1552. 26470294 10.1093/jee/tov185

[mpp13113-bib-0073] Wosula, E.N., McMechan, A.J., Knoell, E., Tatineni, S., Wegulo, S.N. & Hein, G.L. (2018) Impact of timing and method of virus inoculation on the severity of wheat streak mosaic in a virus resistant and susceptible winter wheat cultivar. Plant Disease, 102, 645–650. 30673479 10.1094/PDIS-08-17-1227-RE

[mpp13113-bib-0074] Wosula, E.N., McMechan, A.J., Oliveira‐Hofman, C., Wegulo, S.N., Hein, G.L. (2016) Differential transmission of two isolates of Wheat streak mosaic virus by five wheat curl mite populations. Plant Disease, 100, 154–158. 30688577 10.1094/PDIS-03-15-0342-RE

[mpp13113-bib-0075] Yu, C., Karlin, D.G., Lu, Y., Wright, K., Chen, J. & MacFarlane, S. (2013) Experimental and bioinformatic evidence that raspberry leaf blotch emaravirus P4 is a movement protein of the 30K superfamily. Journal of General Virology, 94, 2117–2128. 10.1099/vir.0.053256-0 23761405

[mpp13113-bib-0076] Zhang, S., Yang, L., Ma, L., Tian, X., Li, R., Zhou, C. et al. (2020) Virome of *Camellia japonica*: discovery of and molecular characterization of new viruses of different taxa in camellias. Frontiers in Microbiology, 11, 945. 32499772 10.3389/fmicb.2020.00945 PMC7243478

[mpp13113-bib-0077] Zheng, Y., Navarro, B., Wang, G., Wang, Y., Yang, Z., Xu, W. et al. (2017) Actinidia chlorotic ringspot‐associated virus: a novel emaravirus infecting kiwifruit plants. Molecular Plant Pathology, 18, 569–581. 27125218 10.1111/mpp.12421 PMC6638214

